# Differential Gut Microbiota Compositions Related With the Severity of Major Depressive Disorder

**DOI:** 10.3389/fcimb.2022.907239

**Published:** 2022-07-11

**Authors:** Qi Zhong, Jian-jun Chen, Ying Wang, Wei-hua Shao, Chan-juan Zhou, Peng Xie

**Affiliations:** ^1^Institute of Life Sciences, Chongqing Medical University, Chongqing, China; ^2^Department of Rehabilitation, The Second Affiliated Hospital of Chongqing Medical University, Chongqing, China; ^3^Department of Respiratory and Critical Care Medicine, The First Affiliated Hospital of Chongqing Medical University, Chongqing, China; ^4^Central Laboratory, Yongchuan Hospital of Chongqing Medical University, Chongqing, China; ^5^Department of Neurology, The First Affiliated Hospital of Chongqing Medical University, Chongqing, China; ^6^NHC Key Laboratory of Diagnosis and Treatment on Brain Functional Diseases, The First Affiliated Hospital of Chongqing Medical University, Chongqing, China

**Keywords:** major depressive disorder, gut microbiota, Firmicutes, Actinobacteriota, Bacteroidota

## Abstract

**Objective:**

Increasing evidence shows a close relationship between gut microbiota and major depressive disorder (MDD), but the specific mechanisms remain unknown. This study was conducted to explore differential gut microbiota compositions related to the severity of MDD.

**Methods:**

Healthy controls (HC) (n = 131) and MDD patients (n = 130) were included. MDD patients with Hamilton Depression Rating Scale (HDRS) score <25 and ≥25 were assigned into moderate (n = 72) and severe (n = 58) MDD groups, respectively. Univariate and multivariate analyses were used to analyze the gut microbiota compositions at the genus level.

**Results:**

Thirty-six and 27 differential genera were identified in moderate and severe MDD patients, respectively. The differential genera in moderate and severe MDD patients mainly belonged to three (Firmicutes, Actinobacteriota, and Bacteroidota) and two phyla (Firmicutes and Bacteroidota), respectively. One specific covarying network from phylum Actinobacteriota was identified in moderate MDD patients. In addition, five genera (*Collinsella*, *Eggerthella*, *Alistipes*, *Faecalibacterium*, and *Flavonifractor*) from the shared differential genera by two MDD groups had a fair efficacy in diagnosing MDD from HC (AUC = 0.786).

**Conclusions:**

Our results were helpful for further exploring the role of gut microbiota in the pathogenesis of depression and developing objective diagnostic methods for MDD.

## Background

Major depressive disorder (MDD) is a common but serious neuropsychiatric disorder that can greatly affect the patients’ quality of life (Martins-de-Souza, 2014; Zhu et al., 2020; Liu et al., 2021; Tian et al., 2021). It is mainly characterized by emptiness or hopelessness, loss of interest, and sleep disturbances (Rana et al., 2021). Previous studies reported that MDD was closely related to hippocampal atrophy, disorder of the hypothalamic–pituitary–adrenal (HPA) axis, and reduction of glial cells in the prefrontal cortex (Ongür et al., 1998; Campbell and Macqueen, 2004; Pariante and Lightman, 2008). However, commonly accepted theories about the pathogenesis of MDD are still not available. Meanwhile, the first-line treatment according to these theories can only alleviate symptoms in about half of MDD patients (Al-Harbi, 2012), and there are no validated biomarkers for objective diagnosis of MDD nowadays. Thus, it is urgently needed to further study the pathogenesis of MDD from new perspectives.

Gut microbiota plays an important role in maintaining the host’s health, and many researchers pay attention to the cross talk between the gut and brain (Han et al., 2020; Qiao et al., 2020; Kovács et al., 2021; Khoshkam et al., 2021). Mounting evidence shows that gut microbiota can affect the host’s brain functions and behaviors *via* the “microbiota–gut–brain” axis (Chen et al., 2021; Rajput et al., 2021; Ding et al., 2021; Dong et al., 2021). In our previous work, we found significant differences in gut microbiota compositions between MDD patients and healthy controls (HC) (Chen et al., 2020; Bai et al., 2021), and these differences were specifically relative to bipolar disorder and schizophrenia (Zheng et al., 2019; Zheng et al., 2020). Other researchers also found that some bacterial taxa, such as *Flavonifractor* and *Faecalibacterium*, changed in patients with depression (Coello et al., 2019; Zhou et al., 2020; Coello et al., 2021). Using an animal depression model, we reported that gut microbiota could induce depression-like behaviors by regulating the host’s metabolism (Zheng et al., 2016) and that glycerophospholipid metabolism might be the vital node between microbiota and depression (Tian et al., 2022). These findings suggested that further exploring the role of gut microbiota in the onset of depression may be helpful for revealing the pathogenesis of MDD.

Many metabolites produced by gut microbiota are closely related to health (Lu et al., 2021; Zhong et al., 2021; Xie et al., 2021). Short-chain fatty acids (SCFAs), as the main products of gut microbiota, have been found to change in many diseases such as cardiovascular disease and autism (Chambers et al., 2018; Tran and Mohajeri, 2021). Our previous studies found some differential microbial metabolites in the urine and plasma of MDD patients (Zheng et al., 2013; Zheng et al., 2013; Chen et al., 2015). Moreover, we found differential urinary and plasma metabolites related to the severity of MDD (Liu et al., 2015; Chen et al., 2017). Considering the close relationships between metabolites and gut microbiota in MDD, we conducted this study to explore whether the differences in gut microbiota compositions were also related with the severity of MDD.

## Methods

### Subject Recruitments

This study was approved by the Ethical Committee of Chongqing Medical University (No. 20200320), and all the included subjects provided written informed consent. Subjects meeting the fourth Diagnostic and Statistical Manual of Mental Disorders criteria for MDD (DSM-IV) were included as MDD patients. HC were from the Medical Examination Center. In total, 131 HC and 130 MDD patients were included from our previous studies (Chen et al., 2020; Bai et al., 2021). There were 21 MDD patients receiving antidepressants (mainly citalopram, fluoxetine, paroxetine, sertraline, and venlafaxine) for 1 month prior to sample collections. In the MDD group, 78 patients with Hamilton Depression Rating Scale (HDRS) score <25 were assigned into the moderate MDD group, and the other 52 patients with HDRS score ≥25 were assigned into the severe MDD group (Kriston and von Wolff, 2011; Liu et al., 2015; Chen et al., 2017). The age, body mass index (BMI), and sex ratio were matched among the three groups. The detailed information of these subjects is found in **Table 1**.

**Table 1:** Characteristics of the included subjects.

	HC	Moderate MDD	*p*-value^a^	Severe MDD	*p*-value^b^	Total MDD	*p*-value^c^
Number	131	78	–	52	–	130	–
Age	37.07 (14.22)	35.77 (13.92)	0.80	37.88 (15.5)	0.93	36.61 (14.57)	0.79
Sex (F/M)	89/42	53/25	0.99	35/17	0.93	88/42	0.96
BMI	21.95 (3.48)	21.76 (2.42)	0.96	21.70 (2.70)	0.94	21.74 (2.52)	0.57
HDRS	0.48 (0.83)	20.47 (2.26)	<0.00001	29.27 (3.70)	<0.00001	23.99 (5.21)	<0.00001
Medication	0/131	13/65	<0.00001	8/44	<0.00001	21/109	<0.00001

^a^p-value was from HC vs. moderate MDD; ^b^p-value was from HC vs. severe MDD; ^c^p-value was from HC vs. total MDD.

HC, healthy controls; MDD, major depressive disorder; F, female; M, male; BMI, body mass index; HDRS, Hamilton Depression Rating Scale.

### Gut Microbiota Compositions

The procedures for the measurement of gut microbiota compositions were identical to our previous studies (Chen et al., 2020; Bai et al., 2021). Briefly, after the raw 16S rRNA gene sequencing reads were obtained using the Illumina MiSeq PE300 platform/NovaSeq PE250 platform (Illumina, San Diego, USA), they were then demultiplexed, quality-filtered by FASTP (version 0.20.0), and merged by FLASH (version 1.2.7) with the following criteria. (i) The 300-bp reads were truncated at any site receiving an average quality score of <20 over a 50-bp sliding window, and the truncated reads shorter than 50 bp were discarded. (ii) Only overlapping sequences longer than 10 bp were assembled according to their overlapped sequence. The maximum mismatch ratio of the overlap region was 0.2. Reads that could not be assembled were discarded. (iii) Exact barcode matching, two-nucleotide mismatch in primer matching, and reads containing ambiguous characters were removed. The operational taxonomic units (OTUs) with 97% similarity cutoff were clustered using UPARSE (version 7.1), and chimeric sequences were removed. The taxonomy of each OTU representative sequence was analyzed by RDP Classifier (version 2.2) against the 16S rRNA database using a confidence threshold of 0.7. At last, we obtained the relative abundances of gut microbiota at different levels. In this study, we analyzed the abundance score for each genus in the three groups.

### Statistical Analysis

Firstly, the Student’s t-test, non-parametric test, chi-square test, or one-way analysis was used to check whether there were significant differences on the demographic data among the three groups (Ma et al., 2021). Secondly, the orthogonal partial least-square discriminant analysis (OPLS-DA) was used to identify the differential genera responsible for the discrimination between MDD patients and HC. Here, the default seven-round cross-validation in OPLS-DA was applied. The genus with important variables on the projection (VIP) > 1.0 (equivalent to a p-value of less than 0.05) was identified as the differential genus. Thirdly, the co-occurrence network was built using the identified differential genera to reflect the microbial changes in HC, moderate MDD patients, and severe MDD patients (Abdullaeva et al., 2021). Fourth, to identify the genera with the promise as the potential biomarkers for diagnosing MDD, the stepwise logistic-regression analysis based on Akaike’s information criterion (AIC) rule was used to analyze the shared differential genera in moderate and severe MDD patients (Ferlizza et al., 2020; Fuchs-Leitner et al., 2021). By dealing with the trade-off between simplicity and goodness of fit of the built model, the AIC rule was often applied to conduct model selection during stepwise logistic-regression analysis (Chen et al., 2020). The model with the minimum AIC value was the preferred model, and genera in this model were viewed as the potential biomarkers. Receiver operating characteristic (ROC) curve analysis was conducted to evaluate the diagnostic performance of the identified potential biomarkers (Kumstel et al., 2020; Wang et al., 2021; Fang et al., 2021). The area under the curve (AUC) was used to assess the diagnostic performance: 0.9–1, excellent; 0.8–0.9, good; 0.7–0.8, fair; 0.6–0.7, poor; and 0.5–0.6, failed. Moreover, sensitivity analysis in the logistic regression model was conducted by excluding these 21 MDD patients receiving antidepressants in 1 month prior to sample collection. Finally, the correlation between HDRS score and the abundance score of all differential genera was investigated. SPSS 19.0 and R software 3.6 were used to do all the analyses, and *P*-value <0.05 was viewed as significant difference.

## Results

### Differential Genera in MDD Patients

**Figure 1:**
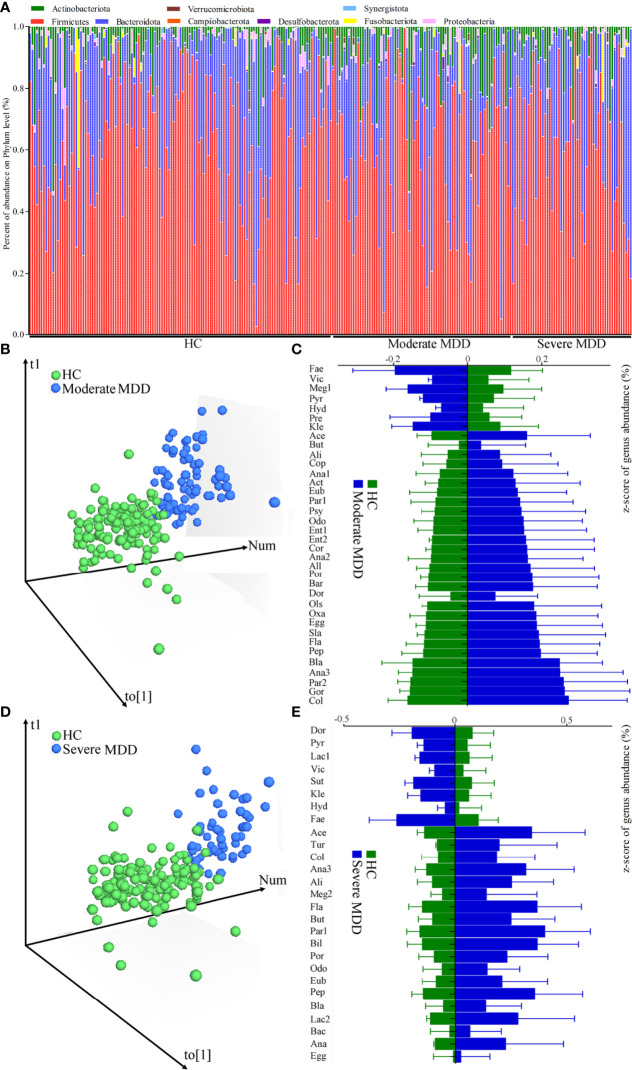
Changes of gut microbiota compositions in HC and moderate and severe MDD patients. **(A)** Relative abundances of gut microbiota at the genus level in MDD patients and HC. **(B)** OPLS-DA model showed that there was only a small overlap between HC and moderate MDD patients, suggesting the divergent microbial changes between the two groups; **(C)** differential genera responsible for discriminating moderate MDD patients from HC; **(D)** OPLS-DA model showed that there was only a small overlap between HC and severe MDD patients, suggesting the divergent microbial changes between the two groups; **(E)** differential genera responsible for discriminating severe MDD patients from HC. HC, healthy controls; MDD, major depressive disorder; Fae, Faecalibacterium; Vic, Victivallis; Meg1, Megamonas; Pyr, Pyramidobacter; Hyd, Hydrogenoanaerobacterium; Pre, Prevotella; Kle, Klebsiella; Ace, Acetanaerobacterium; But, Butyricimonas; Ali, Alistipes; Cop, Coprobacillus; Ana1, Anaerococcus; Act, Actinomyces; Eub, Eubacterium; Par1, Parabacteroides; Psy, Psychrobacter; Odo, Odoribacter; Ent1, Enterococcus; Ent2, Enterorhabdus; Cor, Corynebacterium; Ana2, Anaerofustis; All, Allisonella; Por, Porphyromonas; Bar, Barnesiella; Ols, Olsenella; Dor, Dorea; Oxa, Oxalobacter; Egg, Eggerthella; Sla, Slackia; Fla, Flavonifractor; Pep, Peptoniphilus; Bla, Blautia; Ana3, Anaerotruncus; Par2, Parvimonas; Gor, Gordonibacter; Col, Collinsella; Lac1, Lactococcus; Sut, Sutterella; Tur, Turicibacter; Meg2, Megasphaera; Bil, Bilophila; Lac2, Lactobacillus; Bac, Bacteroides; Ana, Anaeroglobus.

The relative abundances of gut microbiota in MDD patients and HC are described in **Figure 1A**. Firmicutes, Bacteroidota, Actinobacteriota, and Proteobacteria were the main phyla in both MDD patients and HC. Here, two parameters (Shannon and Simpson) were calculated to assess α-diversity, and principal-coordinate analysis was used to assess β-diversity. There was no significant difference on α-diversity between HC and moderate MDD patients (Shannon, p = 0.32; Simpson, p = 0.16), but principal-coordinate analysis showed that there were significant differences on β-diversity between the two groups (p = 0.012). Meanwhile, non-significant differences on α-diversity (Shannon, p = 0.44; Simpson, p = 0.25) and significant differences on β-diversity (p = 0.031) were observed between HC and severe MDD patients.

After adjusting for age, sex, and BMI, the OPLS-DA model displayed that the HC and moderate MDD patients could be obviously separated by the microbiota genera, which suggested the divergent microbial changes between HC and moderate MDD patients (**Figure 1B**). By analyzing the loading plot of the model, 36 genera with VIP > 1.0 were identified as the differential genera in moderate MDD patients. Compared with HC, the abundance scores of *Victivallis*, *Pyramidobacter*, *Hydrogenoanaerobacterium*, *Megamonas*, *Klebsiella*, *Prevotella*, and *Faecalibacterium* were decreased, while those of *Dorea*, *Butyricimonas*, *Alistipes*, *Parabacteroides*, *Blautia*, *Coprobacillus*, *Flavonifractor*, *Odoribacter*, *Actinomyces*, *Collinsella*, *Barnesiella*, *Eubacterium*, *Anaerococcus*, *Allisonella*, *Anaerofustis*, *Oxalobacter*, *Anaerotruncus*, *Acetanaerobacterium*, *Eggerthella*, *Peptoniphilus*, *Enterococcus*, *Gordonibacter*, *Porphyromonas*, *Parvimonas*, *Slackia*, *Psychrobacter*, *Corynebacterium*, *Olsenella*, and *Enterorhabdus* were increased in moderate MDD patients (**Figure 1C**). These differential genera mainly belonged to phyla Firmicutes (n = 16, 44.44%), Actinobacteriota (n = 8, 22.22%), and Bacteroidota (n = 7, 19.44%). The detailed information of these differential genera is described in **Table 2**.

**Table 2:** Differential genera responsible for discriminating moderate MDD patients from HC.

Genus	VIP	FC	Phylum
Enterorhabdus	1.24	0.06	Actinobacteriota
Olsenella	1.34	0.1	Actinobacteriota
Corynebacterium	1.01	0.14	Actinobacteriota
Psychrobacter	1.04	0.15	Proteobacteria
Slackia	1.43	0.17	Actinobacteriota
Parvimonas	1.54	0.19	Firmicutes
Porphyromonas	1.31	0.2	Bacteroidota
Gordonibacter	1.95	0.22	Actinobacteriota
Enterococcus	1.02	0.22	Firmicutes
Peptoniphilus	1.51	0.26	Firmicutes
Eggerthella	1.26	0.28	Actinobacteriota
Acetanaerobacterium	1.07	0.3	Firmicutes
Anaerotruncus	2.11	0.31	Firmicutes
Oxalobacter	1.66	0.31	Proteobacteria
Anaerofustis	1.37	0.32	Firmicutes
Allisonella	1.21	0.32	Firmicutes
Anaerococcus	1.06	0.41	Firmicutes
Eubacterium	1.45	0.42	Firmicutes
Barnesiella	1.37	0.42	Bacteroidota
Collinsella	2.22	0.45	Actinobacteriota
Actinomyces	1.08	0.49	Actinobacteriota
Odoribacter	1.34	0.5	Bacteroidota
Flavonifractor	1.46	0.5	Firmicutes
Coprobacillus	1	0.53	Firmicutes
Blautia	1.83	0.66	Firmicutes
Parabacteroides	1.74	0.67	Bacteroidota
Alistipes	1.47	0.8	Bacteroidota
Butyricimonas	1.53	0.85	Bacteroidota
Dorea	1.02	0.85	Firmicutes
Faecalibacterium	1.19	1.32	Firmicutes
Prevotella	1.24	1.46	Bacteroidota
Klebsiella	1.14	2.39	Proteobacteria
Megamonas	1.19	2.51	Firmicutes
Hydrogenoanaerobacterium	1.39	6.81	Firmicutes
Pyramidobacter	1.46	8.84	Synergistota
Victivallis	1.34	14.11	Verrucomicrobiota

HC, healthy controls; MDD, major depressive disorder; VIP, important variables on the projection; FC, fold change, compared to HC. >1.0 and <1.0 indicated significantly lower and higher levels, respectively, in MDD patients.

Similarly, after adjusting for age, sex, and BMI, using OPLS-DA (**Figure 1D**), we identified 27 differential genera with VIP >1.0 in severe MDD patients. Compared with HC, the abundance scores of *Lactococcus*, *Pyramidobacter*, *Victivallis*, *Sutterella*, *Klebsiella*, *Hydrogenoanaerobacterium*, *Dorea*, and *Faecalibacterium* were decreased, while those of *Eggerthella*, *Bacteroides*, *Blautia*, *Collinsella*, *Odoribacter*, *Alistipes*, *Megasphaera*, *Flavonifractor*, *Butyricimonas*, *Parabacteroides*, *Bilophila*, *Porphyromonas*, *Anaerotruncus*, *Eubacterium*, *Peptoniphilus*, *Acetanaerobacterium*, *Lactobacillus*, *Turicibacter*, and *Anaeroglobus* were increased in severe MDD patients (**Figure 1E**). These differential genera mainly belonged to phyla Firmicutes (n = 14, 51.85%) and Bacteroidota (n = 6, 22.22%). The detailed information of these differential genera is described in **Table 3**.

**Table 3:** Differential genera responsible for discriminating severe MDD patients from HC.

Genus	VIP	FC	Phylum
Anaeroglobus	1.91	0.04	Firmicutes
Turicibacter	1.40	0.09	Firmicutes
Lactobacillus	1.68	0.11	Firmicutes
Acetanaerobacterium	1.61	0.16	Firmicutes
Peptoniphilus	1.66	0.16	Firmicutes
Eubacterium	1.75	0.30	Firmicutes
Anaerotruncus	1.71	0.35	Firmicutes
Porphyromonas	1.37	0.39	Bacteroidota
Bilophila	2.55	0.40	Desulfobacterota
Parabacteroides	2.14	0.41	Bacteroidota
Butyricimonas	1.92	0.43	Bacteroidota
Flavonifractor	2.42	0.43	Firmicutes
Megasphaera	1.00	0.45	Firmicutes
Alistipes	1.83	0.60	Bacteroidota
Odoribacter	1.49	0.65	Bacteroidota
Collinsella	1.47	0.66	Actinobacteriota
Blautia	1.58	0.80	Firmicutes
Bacteroides	1.55	0.90	Bacteroidota
Eggerthella	1.23	0.92	Actinobacteriota
Faecalibacterium	1.41	1.37	Firmicutes
Dorea	1.53	1.55	Firmicutes
Hydrogenoanaerobacterium	1.15	1.98	Firmicutes
Klebsiella	1.37	2.28	Proteobacteria
Sutterella	1.24	2.97	Proteobacteria
Victivallis	1.14	6.39	Verrucomicrobiota
Pyramidobacter	1.27	36.23	Synergistota
Lactococcus	1.03	37.70	Firmicutes

HC, healthy controls; MDD, major depressive disorder; VIP, important variables on the projection; FC, fold change, compared to HC. >1.0 and <1.0 indicated significantly lower and higher levels, respectively, in MDD patients.

### Co-occurrence Network of Differential Genera

**Figure 2:**
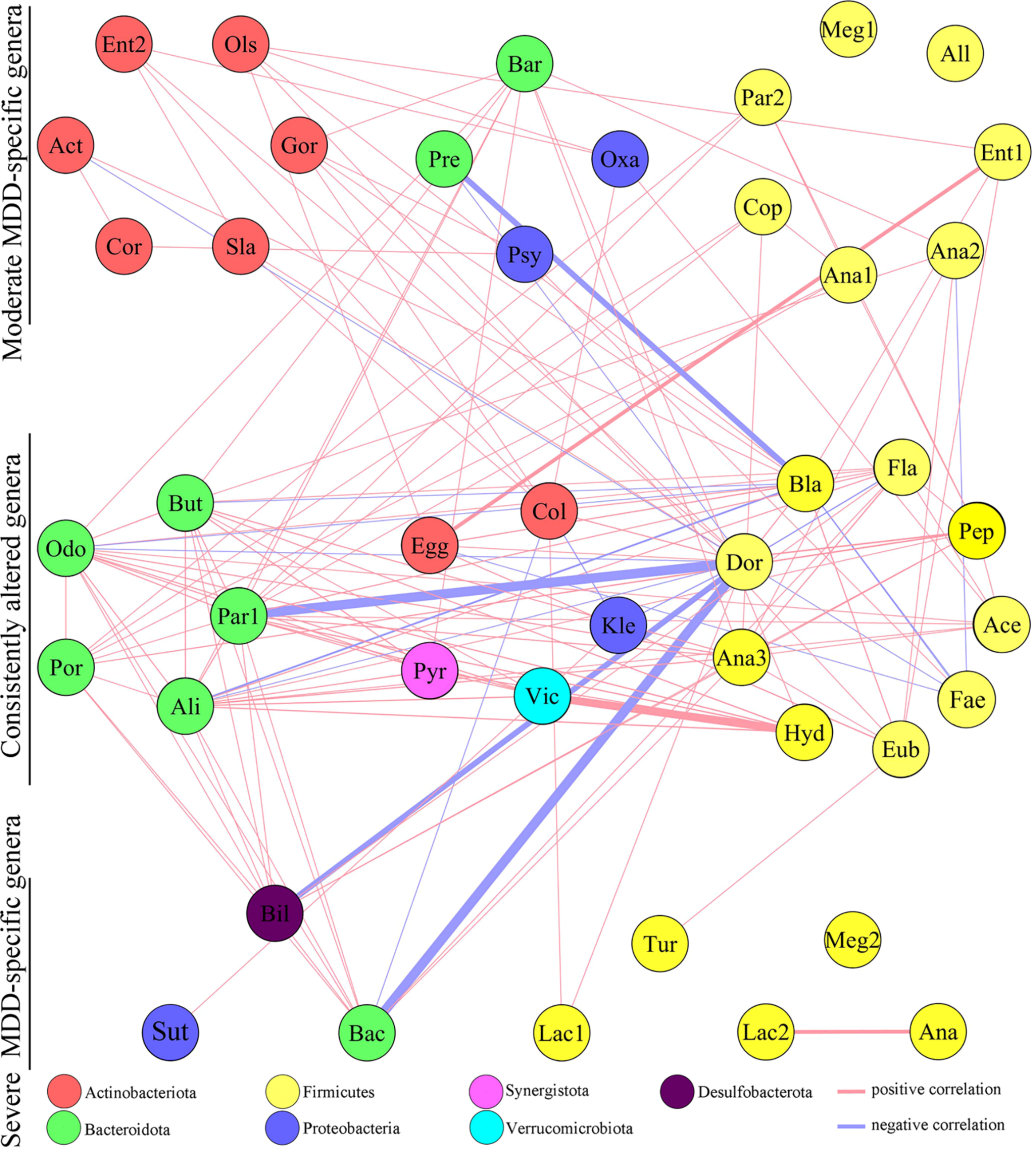
Co-occurrence network showing microbial changes in moderate and severe MDD patients. The microbial genera changed in moderate or severe MDD were identified by OPLS-DA. In total, 63 differential genera were identified in the two groups. Nineteen of 63 genera were consistently altered in both moderate and severe MDD patients relative to HC, and 17 and 8 genera were specific to moderate MDD alone and severe MDD alone, respectively. Compared to HC, moderate MDD was mainly characterized by altered covarying genera assigned to phylum Firmicutes, Actinobacteriota, and Bacteroidota, while severe MDD was mainly characterized by altered covarying genera assigned to phyla Firmicutes and Bacteroidota. Lines between nodes indicate Spearman’s correlation > +0.30 (light red) or < −0.30 (light blue)); line thickness indicates p value (p < 0.05).

The co-occurrence networks deduced from the relative abundance of moderately or severely related genera were generated using Spearman’s correlation coefficient, which was used to reflect microbial changes in HC and moderate and severe MDD patients. As shown in **Figure 2**, the majority of altered genera belonging to phylum Actinobacteriota (n = 6, 75%) were specific to moderate MDD patients. The co-occurrence network showed that in moderate MDD patients, six genera from phylum Actinobacteriota and seven genera from phylum Firmicutes significantly covaried with one another, which generated two characteristic covarying networks from phyla Actinobacteriota and Firmicutes. No such specific covarying network was found in severe MDD patients. Meanwhile, among the identified differential genera, 19 (nine belonging to phylum Firmicutes and five belonging to phylum Bacteroidota) were consistently changed in MDD patients compared with HC (**Figure 2**). The co-occurrence network showed that there were two characteristic covarying networks from phyla Bacteroidota and Firmicutes in MDD groups.

### Potential Biomarkers for *Diagnosing* MDD

**Figure 3:**
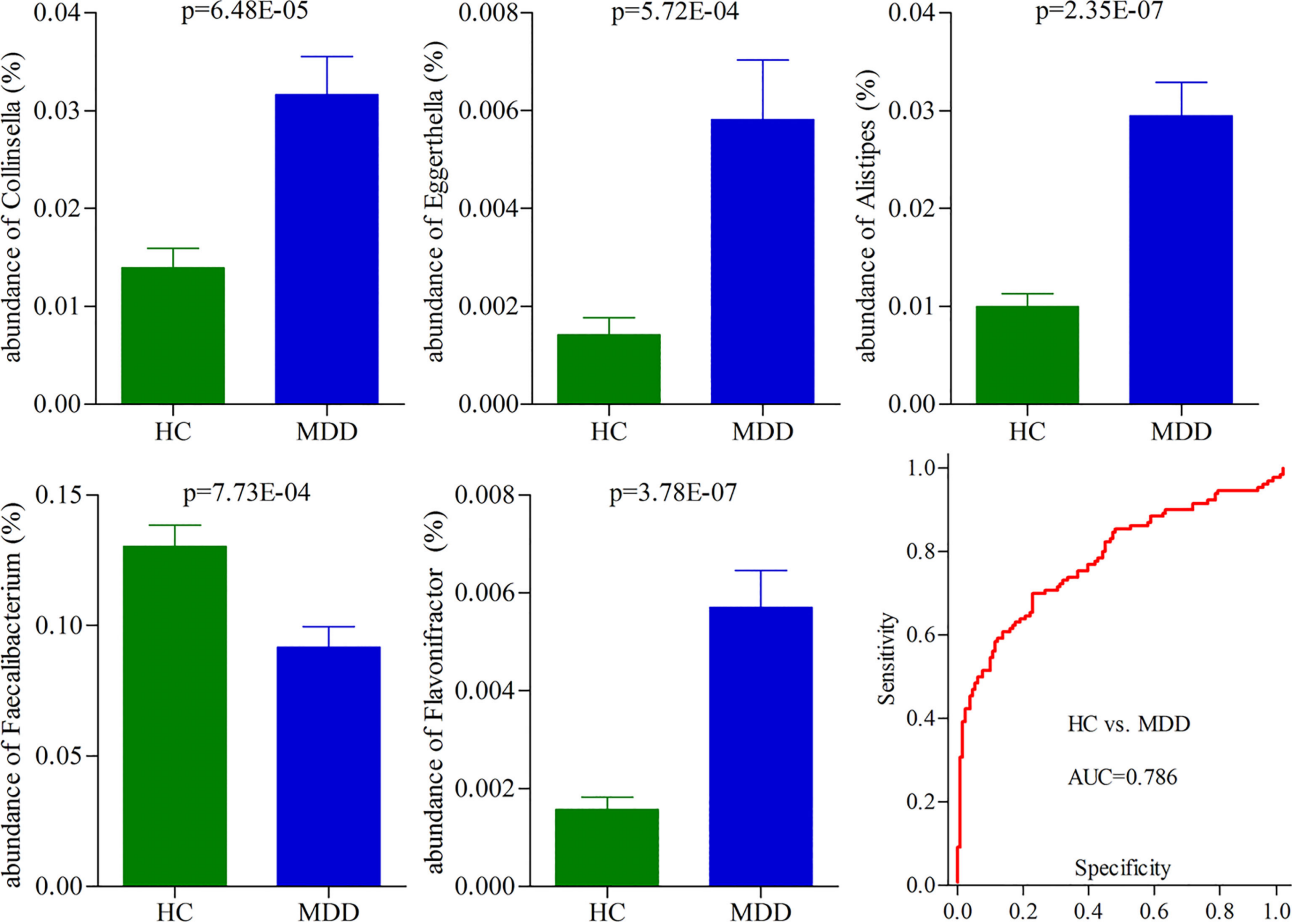
Five differential genera as potential biomarkers for diagnosing MDD. The model consisting of these five genera had the minimum AIC value; thus, they were viewed as the potential biomarkers. The panel consisting of these five genera could yield an AUC of 0.786 for classifying MDD patients from HC, suggesting fair diagnostic performance in diagnosing MDD. HC, healthy controls; MDD, major depressive disorder; AUC, area under the curve.

To identify the potential biomarkers for diagnosing MDD, we used the logistic regression analysis to further analyze the consistently changed genera in both moderate and severe MDD patients when compared with HC. After adjusting for age, sex, and BMI, the results showed that the most significant deviations between HC and MDD patients were explained by five differential genera (*Collinsella*, *Eggerthella*, *Alistipes*, *Faecalibacterium*, and *Flavonifractor*) (**Figure 3**). The ROC curve analysis was then used to evaluate the diagnostic performance of these differential genera. The results showed that the panel consisting of these five genera could yield an AUC of 0.786 for classifying MDD patients from HC (**Figure 3**). The sensitivity analysis by excluding the medicated MDD patients showed a similar diagnostic performance of this panel in diagnosing MDD. These results suggested that these five differential genera might hold promise as potential biomarkers for diagnosing MDD.

### Correlation Between HDRS and Differential Genera

**Figure 4:**
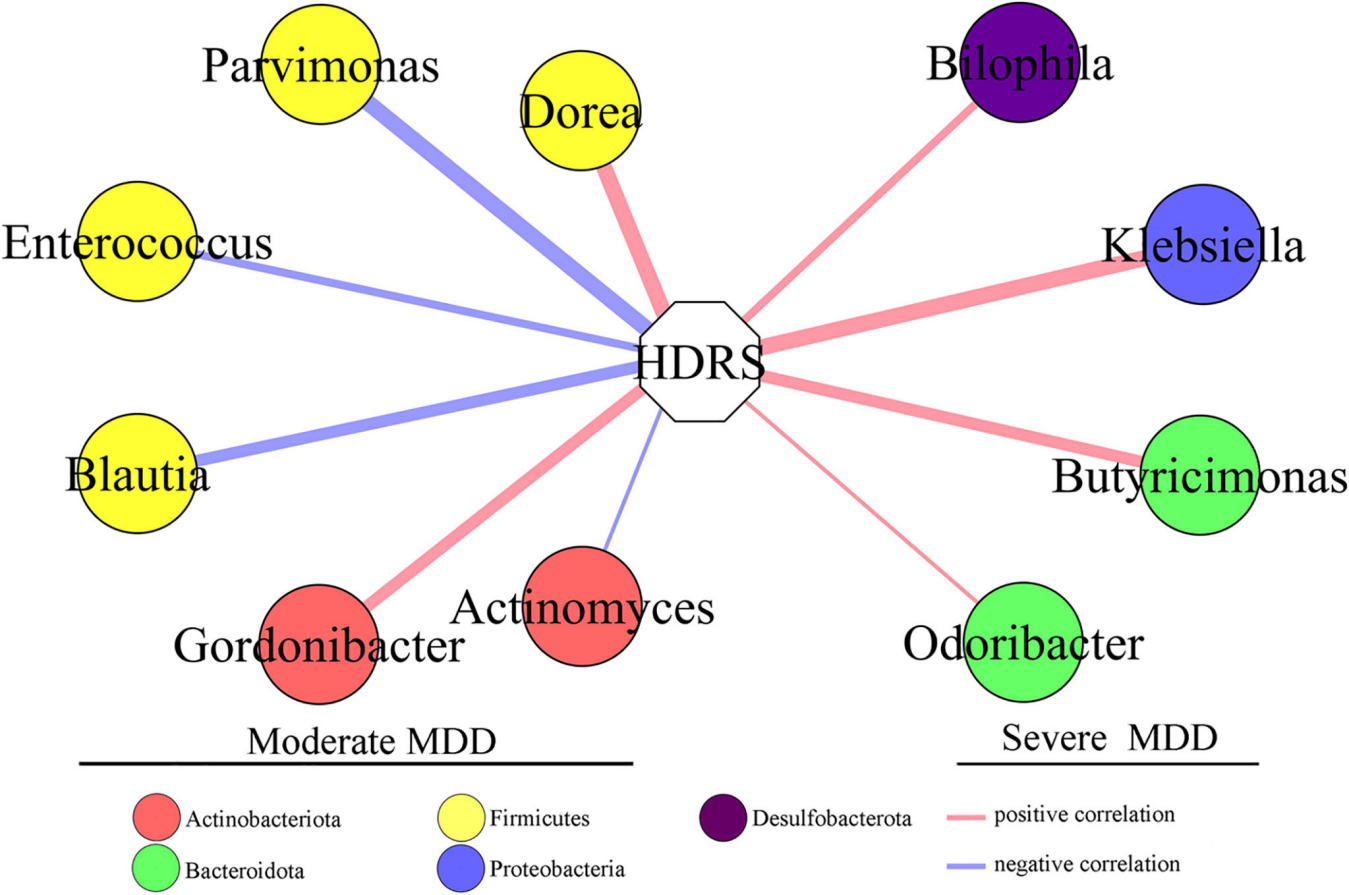
Differential genera in moderate and severe MDD patients significantly correlated with HDRS. Six genera (four of them belonged to phylum Firmicutes) in moderate MDD patients were significantly positively or negatively correlated with HDRS. Four genera in severe MDD patients were significantly positively correlated with HDRS. MDD, major depressive disorder; HDRS, Hamilton Depression Rating Scale.

To find out the potential correlations between the severity of depression and gut microbiota, Pearson correlation analysis was used to analyze the correlations between HDRS score and differential genera. The genera significantly correlated with the HDRS score were used to build the correlation network (**Figure 4**). Six differential genera (*Parvimonas*, *Dorea*, *Gordonibacter*, *Blautia*, *Actinomyces*, and *Enterococcus*) in moderate MDD patients presented significantly positive or negative correlations with the HDRS score. Four differential genera (*Klebsiella*, *Butyricimonas*, *Bilophila*, and *Odoribacter*) in severe MDD patients presented significantly positive correlations with HDRS.

### Moderate MDD vs. Severe MDD

**Figure 5:**
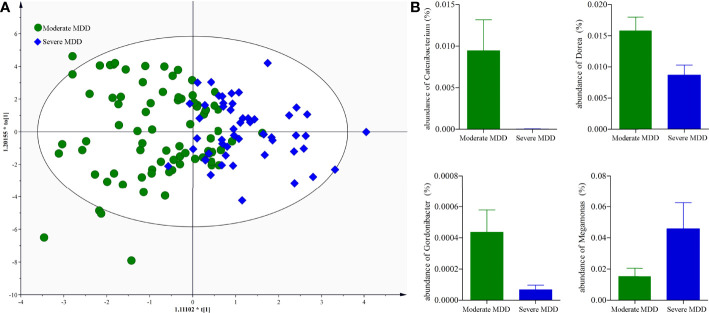
Genus-level analysis of gut microbiota between moderate and severe MDD patients. **(A)** OPLS-DA model showed that the moderate and severe MDD patients could not be significantly separated; **(B)** there were four differential genera between the two groups.

It might be interesting to see the differential genera between moderate and severe MDD patients; thus, we directly used the data from moderate and severe MDD patients to build the OPLS-DA model. Results showed no significant difference on both α-diversity (Shannon, p = 0.51; Simpson, p = 0.47) and β-diversity (p = 0.22) between moderate and severe MDD patients. Meanwhile, after adjusting for age, sex, and BMI, the built OPLS-DA model displayed that moderate and severe MDD patients could not be clearly separated (40.38% severe MDD patients were wrongly assigned into moderate MDD patients) (**Figure 5A**). However, we still identified four differential genera (*Catenibacterium*, VIP = 1.73; *Dorea*, VIP = 2.16; *Gordonibacter*, VIP = 1.45; *Megamonas*, VIP = 1.58) between moderate and severe MDD patients. Compared with severe MDD patients, Catenibacterium, Dorea, and Gordonibacter were significantly higher, while *Megamonas* was significantly lower in the moderate MDD patients (**Figure 5B**).

## Discussion

This study was conducted to find the divergent microbes of different MDD severity. The results showed that there were 36 and 27 differential genera in moderate and severe MDD patients, respectively. The differential genera in moderate and severe MDD patients mainly belonged to three (Firmicutes, Actinobacteriota, and Bacteroidota) and two phyla (Firmicutes and Bacteroidota), respectively. Meanwhile, one specific covarying network from phylum Actinobacteriota was identified in moderate MDD patients. In addition, the moderate and severe MDD patients shared no differential genera that were significantly correlated with the HDRS score. Therefore, these findings suggested that although moderate and severe MDD patients shared some common differential genera, the two groups had significantly different microbial signatures.

Currently, clinicians still use the structured clinical interview rather than objective laboratory tests to diagnose MDD. However, the interview method often results in a certain percentage of misdiagnosis (Mitchell et al., 2009) due to the highly heterogeneous of clinical presentation of MDD. One promising way to markedly increase the accuracy of diagnosis is to identify disease biomarkers for objectively diagnosing MDD. In recent decades, much work has been done to identify potential biomarkers for MDD (Dmitrzak-Weglarz et al., 2021; Bai et al., 2021; Huang et al., 2022; Travica et al., 2022). However, few studies have taken the severity of MDD into consideration. In our previous studies, the differential urinary and plasma metabolites related to the severity of MDD were observed (Liu et al., 2015; Chen et al., 2017). Here, we provided an interesting method to identify potential biomarkers for MDD. A panel consisting of five consistently changed genera was found to have fair efficacies in diagnosing MDD patients from HCs.

Gut microbiota could be influenced by many factors, such as dietary habit and antibiotic agents (Farag et al., 2020; Khan et al., 2020; Dordević et al., 2021; Liu et al., 2021). Madison et al. reported that dietary habit could affect the gut microbiota compositions independently or in conjunction with stress (Madison and Kiecolt-Glaser, 2019). Lv et al. found that there was a close relationship between BMI and gut microbiota compositions in Chinese male college students (Lv et al., 2019). Duan et al. observed that the gut microbiota compositions were different in cynomolgus macaques with different ages (Duan et al., 2019). Our previous study found the differential gut microbiota compositions between young and middle-aged MDD patients (Chen et al., 2020). In the present study, our findings further suggested that the gut microbiota compositions could also be affected by the severity of MDD. These results might provide a novel clue for understanding the role of gut microbiota in the onset of depression. However, only gut microbiota at the genus level was analyzed here. Therefore, our identified potential microbial biomarkers—although very promising—were preliminary results and need further validation.

Firmicutes and Bacteroidetes are the two phyla of dominating bacteria in human gut microbiota. Zheng et al. found that the relative abundance of Bacteroidetes was significantly changed in MDD patients compared with HCs (Zheng et al., 2016). Jiang et al. reported that both the relative abundances of Firmicutes and Bacteroidetes were significantly disordered in MDD patients compared with HCs (Jiang et al., 2015). In our previous study, we found that compared with HCs, the relative abundance of Bacteroidetes was significantly increased and decreased in young and middle-aged MDD patients, respectively, and the relative abundance of Firmicutes was only found to be significantly changed in young MDD patients (Chen et al., 2020). In this study, we observed that the differential genera in moderate and severe MDD patients mainly belonged to three (Firmicutes, Actinobacteriota, and Bacteroidota) and two (Firmicutes and Bacteroidota) phyla, respectively. These results indicated that the gut microbiota compositions could be affected by many factors, and further studies on the associations between MDD and gut microbiota should minimize the influence of confounding factors.

The shared differential genus *Collinsella* by two MDD groups is an important intestinal bacterium to produce ursodeoxycholic acid. Ursodeoxycholic acid has antioxidant and anti-apoptotic effects and can suppress pro-inflammatory cytokines like IL-2 and TNF-α (Hirayama et al., 2021). The close relationships between MDD and inflammation have been reported in many previous studies (Leonard, 2018; Colasanto et al., 2020). Another shared differential genus *Faecalibacterium* is an important intestinal bacterium to produce butyric acid. Butyric acid is a major short-chain fatty acid (SCFA) produced by gut microbiota (Sun et al., 2021). SCFAs are speculated to play an important role in the cross talk between the gut and brain. Our previous study found associations between disordered hypothalamus neurotransmitters and fecal SCFAs in depressed mice (Wu et al., 2020). These results showed that the identified shared differential genus were worthy of further exploring.

Previous studies reported that the dominant taxa were different in the different phases of the life cycle (Lim et al., 2015; Vemuri et al., 2018). Our previous study found that there were age-specific differential changes on gut microbiota composition in MDD patients (Chen et al., 2020). In this study, we identified three significantly decreased and one significantly increased genus in severe MDD patients compared with moderate MDD patients. Three of them (*Catenibacterium*, *Dorea*, *Megamonas*) belonged to the phylum Firmicutes. These results indicated that the continuing changes of gut microbiota in moderate MDD patients, especially phylum Firmicutes, might contribute to the deterioration of depression. Therefore, developing personalized treatment methods to timely treat moderate MDD patients might be able to alleviate or delay the progress of depression.

Many studies have reported the microbial markers of depression (Jiang et al., 2015; Chen et al., 2018; Zhou et al., 2020; Yang et al., 2020). Zhou et al. found that gut microbiota-based biomarkers, such as *Faecalibacterium* and *Butyricicoccus*, might be helpful for the diagnosis and treatment of postpartum depressive disorder patients (Zhou et al., 2020). Here, *Faecalibacterium* was also identified as a potential biomarker for MDD. Another study reported that a combinatorial marker panel consisting of bacterial species and fecal metabolite markers could effectively discriminate MDD from HC (Yang et al., 2020). Our previous study found that the suitability of Actinobacteria and Bacteroidia as the sex-specific biomarkers for diagnosing MDD was worthy of further exploring (Chen et al., 2018). Jiang et al. observed that *Alistipes* and *Faecalibacterium* might be potential biomarkers for MDD patients (Jiang et al., 2015). Here, *Collinsella* and *Eggerthella* belonged to phylum Actinobacteriota and *Alistipes* belonged to phylum Bacteroidota were identified as potential biomarkers for MDD. Although these results showed a potential and novel method for objective diagnosis of depression, further studies were warranted to evaluate the suitability of gut microbiota as a biomarker for depression.

Several limitations should be mentioned here. Firstly, the number of subjects in each group was relatively small, which requires future studies to validate and support the conclusions. Secondly, although the potential effects of main confounding factors (age, BMI, sex ratio) were eliminated, the effects of other potential factors, such as family history of psychiatric diseases, host genetics, smoking, and dietary habit, were not explored here; thus, future studies were needed to assess the effects of these factors. Thirdly, all the included subjects came from the same place, and thus there might be ethno-specific biases, which could limit the applicability of our conclusion. Fourthly, due to technical reasons, the identification of gut microbiota at the species level was unsuccessful. Therefore, it might also be meaningful to further investigate the differential gut microbiota compositions at the species level. Fifthly, we did not analyze the functions of differential gut microbiota related to the severity of MDD, which was worthy of further exploring using whole-genome sequencing (WGS) or phylogenetic investigation of communities by reconstruction of unobserved states (PICRUST). Sixthly, the “healthy human microbiota” is only a theoretical phenomenon. Due to the complexity of assessing the health status of gut microbiota, the “healthy human microbiota” has not yet been defined. Thus, the microbial biomarkers should be cautiously interpreted. Seventhly, although the sensitivity analysis showed that the results obtained by excluding these 21 MDD patients were similar to the original results, we did not know whether 1 month was enough to remove the effects of antidepressive treatments on gut microbiota; thus, future studies should recruited drug-naïve MDD patients to evaluate our results.

## Conclusion

In conclusion, this study found that there were divergent microbial phenotypes between moderate and severe MDD patients. Totally, 36 and 27 differential genera in moderate and severe MDD patients, respectively, were identified. One specific covarying network from phylum Actinobacteriota was identified in moderate MDD patients. In addition, five differential genera (Collinsella, Eggerthella, Alistipes, Faecalibacterium, and Flavonifractor) held promise as the potential biomarkers for diagnosing MDD. Our results may also be helpful for further exploring the role of gut microbiota in the pathogenesis of depression.

## Data Availability Statement

The datasets presented in this study can be found in online repositories. The names of the repository/repositories and accession number(s) can be found below: https://www.ncbi.nlm.nih.gov/, PRJNA806486.

## Ethics Statement

The studies involving human participants were reviewed and approved by Ethical Committee of Chongqing Medical University. The patients/participants provided their written informed consent to participate in this study.

## Author Contributions

QZ, J-JC, and PX conceived and designed the study; QZ, YW, and W-HS participated in data collection. J-JC and C-JZ analyzed the data. QZ, J-JC, and PX prepared the paper. All authors have read and approved the final manuscript.

## Funding

This work was supported by the National Key Research and Development Program of China (2017YFA0505700), the Non-profit Central Research Institute Fund of Chinese Academy of Medical Sciences (2019PT320002), the Natural Science Foundation Project of China (81820108015, 81701360), the Natural Science Foundation of Chongqing (cstc2021jcyj-msxmX0084), the Science and Technology Research Program of Chongqing Municipal Education Commission (Grant No. KJQN202100420), and the Chongqing Yuzhong District Science & Technology Commission (20190115).

## Conflict of Interest

The authors declare that the research was conducted in the absence of any commercial or financial relationships that could be construed as a potential conflict of interest.

## Publisher’s Note

All claims expressed in this article are solely those of the authors and do not necessarily represent those of their affiliated organizations, or those of the publisher, the editors and the reviewers. Any product that may be evaluated in this article, or claim that may be made by its manufacturer, is not guaranteed or endorsed by the publisher.
